# Serpins in the Spotlight: Novel Bioinformatic Insights Into *Hyalomma dromedarii* Sialotranscriptome

**DOI:** 10.1155/bri/1309981

**Published:** 2025-12-05

**Authors:** Hajer Aounallah, Ahmed Ouni, Fernanda Faria, Ali Bouattour, Youmna M’ghirbi

**Affiliations:** ^1^ Laboratory of Viruses, Vectors and Hosts (LR20IPT02), Pasteur Institute of Tunis, University of Tunis El Manar, Tunis, Tunisia, utm.rnu.tn; ^2^ Development and Innovation Laboratory, Development and Innovation Center, Butantan Institute, São Paulo, Brazil, butantan.gov.br

**Keywords:** bioinformatics, *Hyalomma dromedarii*, serpins, sialotranscriptome

## Abstract

Ticks pose a significant global threat to human and animal health as vectors of numerous pathogens, including bacteria, viruses, and parasites. Beyond their harmful impact, tick salivary glands contain serine protease inhibitors (serpins) known for their potential pharmaceutical properties. Traditional methods for studying tick serpins are labor‐intensive, but recent advancements in bioinformatics have enabled comprehensive analyses of these molecules. In this study, we employed in silico tools to identify, classify, and analyze serpins encoded within the sialotranscriptome of the camel tick, *Hyalomma dromedarii.* Through sequence analysis, conserved motifs and domains have been identified, shedding light on evolutionary relationships and functional conservation among serpins both within and between tick species. The complexity of *H. dromedarii* serpins (HDS) exceeded prior expectations, with the identification of 15 transcripts exclusively expressed in male and 4 transcripts in female *H. dromedarii* salivary glands, while 91 transcripts are common to both genders. Interestingly, each HDS has a distinct reactive center loop (RCL) sequence at the protein sequence level. These RCL sequences extend from P17 to P4′, are similar to those of other serpins, comprise 21 amino acids, and are situated near the C‐terminus. All RCLs feature a diverse array of eight amino acid residues at the P1 sites, with the majority (47.06%) having polar basic residues. Moreover, our predictions imply that some HDSs may exert regulatory control over a wide array of proteolytic pathways, indicating their potential involvement in modulating numerous biological processes. Overall, our findings provide valuable insights into tick serpins and lay a solid foundation for future research aiming to exploit these molecules for the development of novel therapeutics and vaccines against tick‐borne diseases.

## 1. Introduction

Serine protease inhibitors (serpins) represent one of the broadest superfamilies of protease regulators, with members identified across all biological kingdoms [1]. The name “serpin” was coined by two of the pioneers in the field, Robin Carrell and Jim Travis, owing to their ability to inhibit serine proteases [2]. Nevertheless, this name is somewhat misleading, since certain serpins have been demonstrated to inhibit other type of enzymes such as papain‐like cysteine proteases [3], metalloproteases, and caspases [4]. While their biochemical roles in coagulation, inflammation, and immune regulation are well documented [5], their genomic diversity, domain evolution, and structure–function relationship remain poorly resolved from a computational perspective, particularly in nonmodel hematophagous arthropods.

Ticks, as long‐term blood‐feeding ectoparasites, have evolved a sophisticated salivary secretome enriched with diverse bioactive molecules that modulate host defense systems [6–9]. Indeed, the intricate relationship between ticks and their hosts, woven through millions of years of coevolution, encompasses a myriad of molecular interactions crucial for tick survival and reproduction [6]. Serpins stand at the forefront of this relationship, playing pivotal roles in modulating host hemostasis [10], immune responses [11, 12], inflammation [13], complement system [14], and pathogen transmission [11, 15]. Despite their significance, our understanding of tick serpins remains fragmented, with many aspects of their function and regulation shrouded in mystery. To date, most studies have relied on classical biochemical characterization of individual serpins, leaving a major computational gap in large‐scale sequence clustering, phylogenomic reconstruction, structural modeling, and target prediction of tick‐derived serpin families. So far, several tick salivary serpins have been tested in vitro and in vivo for their therapeutic potential, with the most effective ones currently undergoing preclinical and clinical trials [13]. However, plenty of cDNA‐coding tick serpins remain uncharacterized and are worthy of a thorough investigation.

Compared to other tick species, there have been a limited number of studies investigating serpins from *Hyalomma dromedarii* (Koch 1844), the most prevalent tick in southern Tunisia [16, 17]. Recent transcriptomic and proteomic resources have revealed the presence of multiple *H. dromedarii* serpins (HDSs) in sialotranscriptome [18] and sialoproteome datasets [19]. To date, only a single comprehensive functional characterization of an individual recombinant salivary serpin, termed Dromaserpin, has been reported, demonstrating its anticoagulant and antiplatelet aggregation activities in vitro [10]. However, a systematic in silico functional prediction of HDSs, integrating gene architecture, structural conservation, and host‐interaction potential, has not yet been undertaken. Moreover, computational approaches for reactive center loop (RCL) amino acid profiling, P1 residue identification, and prediction of host protease specificity remain absent in the current tick serpin literature, representing a critical gap for bioinformatic annotation.

In this study, we present the first integrative computational characterization of the serpin repertoire of *H. dromedarii*, combining multiomics data mining and comparative analyses across tick species. We systematically compare HDS with orthologous serpin families from other hematophagous ticks, both within and across species boundaries, and perform in‐depth sequence and functional annotation to predict their potential host targets. Beyond descriptive cataloging, this work establishes a computational framework that accelerates serpin annotation in vector biology and generates a ranked set of *Hyalomma* serpins with high‐confidence predictions for hemostasis modulation and pathogen transmission, guiding candidates for downstream experimental validation.

## 2. Materials and Methods

### 2.1. Ethical Approval

The study was approved by the Commission on Ethics and Animal Welfare of the Institute Pasteur of Tunis‐University of Tunis El Manar, Tunisia, with the given number IPT/LR03/Project PCI/25/2012. All technical procedures were in accordance with the National and the European Legislation regarding animal welfare and have met the International Guiding Principles for Biomedical Research Involving Animals by the Council for the International Organizations of Medical Sciences.

### 2.2. Sequence Analysis of HDS Transcripts

The HDS transcripts analyzed in this study were acquired through data mining of *de novo* assembled *H. dromedarii* sialotranscriptome [18]. The sialotranscriptome was constructed from HiSeq 1500 Illumina sequence reads (BioProject accession number PRJNA358517), originating from partially engorged female and male ticks at various nearly feeding stages. The methodological details of transcriptome construction have been described previously [18], and the raw data were used here to specifically identify HDS transcripts.

Initially, the TransDecoder utility was employed to predict open reading frames (ORFs) from the assembled transcripts. Predicted amino acid sequences were subsequently annotated and validated through BLASTp searches against the up‐to‐date 2024 versions of the UniPro‐tKB/TrEMBL, Uniprot‐Acari, Uniprot‐SwissProt, Pfam database, Eukaryotic Orthologous Groups (KOGs), and NR‐NCBI. BLASTp results were filtered to retain only the top hit for each query. The estimation of transcript abundance was determined using various metrics, including transcripts per million (TPM), fragments per kilobase million (FPKM), isoform percentage (IsoPct), and the number of reads per transcript. These metrics were employed to assess differential gene expression between male and female ticks. Transcripts exhibiting zero TPM values were deemed nonexpressed in the respective gender.

### 2.3. Identification and Comparative Sequence Analysis Among HDS Sequences

The identification of putative HDS sequences involved two sequential steps. Firstly, annotated amino acid sequences underwent filter screening to identify sequences predicted as putative serpins based on their alignment with the mentioned databases. Subsequently, transcript sequences associated with the serpin superfamily were employed to predict the full‐length serpin sequence using the Expasy Translate tool (https://web.expasy.org/translate/). The ScanProsite tool (https://prosite.expasy.org/scanprosite/) was then utilized to identify the serpin signature motif PS00284 in the resulting amino acid sequences.

Validation of putative serpins involved manual inspection to confirm the presence of two consensus amino acid motifs: the RCL in the C‐terminus, based on the eight‐residue pattern “p17 [E]‐p16 [E/K/R]‐p15 [G]‐p14 [T/S]‐p13 [X]‐p12‐9 [AGS]‐p8‐1 [X]‐p1’ ‐4′” and the “NAVYFKG” motif in the N‐terminus. Each serpin received an HDS number and was categorized into two groups: (i) full‐length HDSs and (ii) partial HDSs. Sequences with a starting methionine, ranging from 350 to 450 amino acids long and featuring a unique RCL sequence, were considered putatively full‐length. The SignalP 5.0 web server (https://services.healthtech.dtu.dk/services/SignalP-5.0/) was employed to detect signal peptides in full‐length HDSs and ProtParam tool from Expasy (https://web.expasy.org/protparam/) computed their molecular weights. NetNGlyc 1.0 (https://services.healthtech.dtu.dk/services/NetNGlyc-1.0/) and NetOGlyc 4.0 (https://services.healthtech.dtu.dk/services/NetOGlyc-4.0/) servers predicted potential N‐glycosylation and O‐glycosylation sites, respectively. ClustalW (https://www.genome.jp/tools-bin/clustalw (accessed on 10 February 2024)) facilitated the alignment of RCLs of full‐length HDSs, ESPript 3.0 (https://espript.ibcp.fr/ESPript/ESPript/) was used for visualization, and the RCL sequences were then edited manually. Sequences lacking an RCL region were designated as partial in the C‐terminus and were exclusively used to evaluate differential gene expression between genders.

### 2.4. Phylogenetic Analyses of HDSs and Other Tick Serpins

To explore relationships among HDSs and other tick serpins from publicly available databases, multiple sequence alignments were performed using ClustalW algorithm (https://www.genome.jp/tools-bin/clustalw (accessed on 10 February 2024)). The phylogenetic analysis incorporated the entire amino acid sequence at the interspecies level and focused on the RCL region at the intraspecies level. At the whole amino acid sequence level, protein sequences of 29 serpins from tick species were retrieved from GenBank. For both analyses, the maximum likelihood method and JTT matrix‐based model were used to construct guide phylogeny tree [20]. Initial trees for the heuristic search were obtained automatically by applying neighbor‐joining and BioNJ algorithms to a matrix of pairwise distances estimated using the JTT model and then selecting the topology with superior log likelihood value. Human antithrombin III was used as outgroup to root the intraspecies tree. The interspecies level was rooted on the midpoint and was edited using the iTOL online service (https://itol.embl.de/). Evolutionary analyses were conducted in MEGA11 [21].

## 3. Results

### 3.1. Male and Female *Hyalomma dromedarii* Ticks Produce Distinct Amount of Serpins

Data analysis of the sialotranscriptome in both male and female *H. dromedarii* ticks revealed notable differences in the expression of serpins. Specifically, the analysis identified 4 (3.64%) HDS sequences that were exclusively expressed in females and 15 (13.64%) HDS sequences exclusive to males. Furthermore, there were 91 (82.72%) HDS sequences found in both genders, indicating a significant overlap in the serpin repertoire between male and female ticks (Figure 1(a)). Surprisingly, male *H. dromedarii* ticks exhibited a higher abundance of serpin transcripts compared to female ticks (106 vs. 95 transcripts). However, when examined on a per‐transcript basis, female transcripts showed significantly higher expression levels compared to males, where they were occasionally minimal. For instance, HDS22 and HDS28 were identified in both genders, yet they were prominently expressed in female and poorly represented in male *H. dromedarii* (Figure 1(b)). By examining the heatmap (Figure 1(b)), we discerned two primary clusters of HDS demonstrating either similar or distinct expression patterns across sexes. Specifically, HDS22, HDS32, HDS21, HDS12, HDS26, HDS20, HDS35, HDS27, and HDS28 were grouped closely and exhibited consistently higher expression in female ticks compared to male ticks. Conversely, another set of HDS (HDS30, HDS31, HDS19, HDS13, HDS15, HDS34, HDS17, and HDS18) is clustered with analogous expression profiles.

Figure 1Gender differences of HDSs expressed in the sialotranscriptome of *Hyalomma dromedarii* tick. (a) Venn diagram illustrating differentially expressed HDSs in female and male *H. dromedarii*. (b) Heatmap showing transcript expression levels (log2 TPM) of the HDSs expressed in female (F) and male (M) *H. dromedarii.* The phylogenetic tree was inferred by using the maximum likelihood method and JTT matrix‐based model [20]. The tree with the highest log likelihood (−11413.26) is shown. Initial tree(s) for the heuristic search were obtained automatically by applying neighbor‐joining and BioNJ algorithms to a matrix of pairwise distances estimated using the JTT model and then selecting the topology with superior log likelihood value. This analysis involved 18 amino acid sequences. There were a total of 481 positions in the final dataset. Evolutionary analyses were conducted in MEGA11 [21]. The numbers on the branches represent branch lengths. Antithrombin III was used as an outgroup to root the tree.

**Figure:**
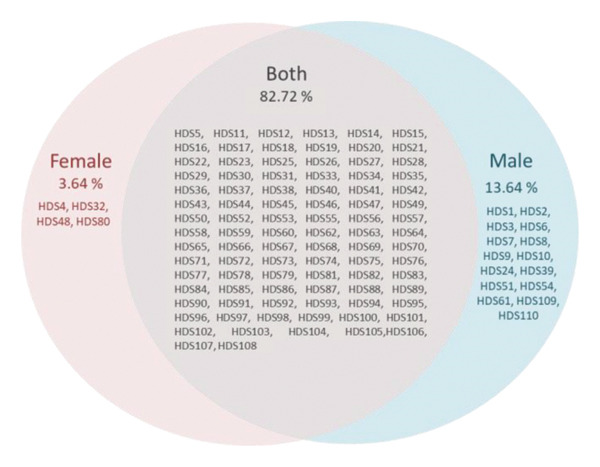
(a)

**Figure:**
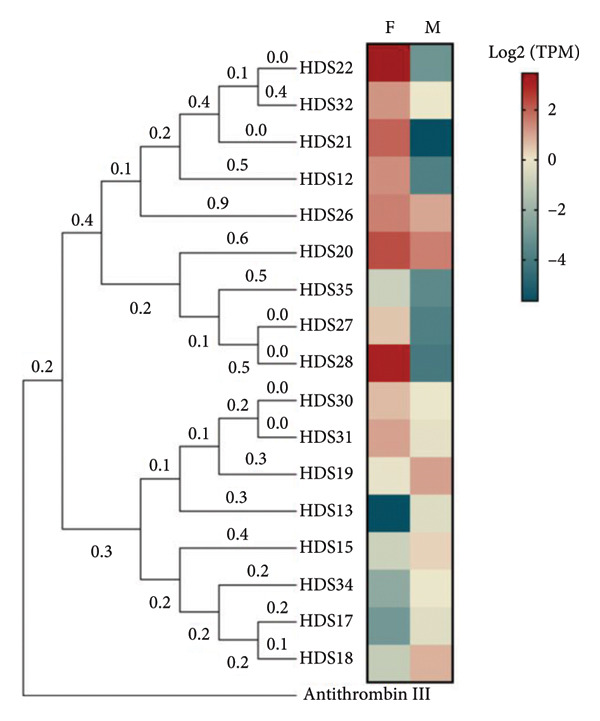
(b)

### 3.2. HDS Sequences Exhibit Typical Serpin Features, With Certain Sequences Showing High Similarities

**Figure 2:**
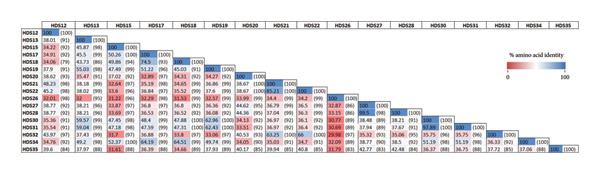
The pairwise comparison of the percentage of similarities between the HDS sequences. The similarities are shown in % amino acid sequence similarities/distances (% coverage) and were inferred from a MUSCLE alignment (standard settings, alignment not shown).

Based on the predicted amino acid sequences of each HDS and after inspection of the presence of consensus motifs, 84.54% of HDSs were found to be partial, truncated at their C‐terminus. Only 15.46% of identified HDSs were found to be complete, containing the consensus serpin amino‐terminus motif (NAVYFKG) and the starting methionine. Each of these complete HDSs featured a unique RCL sequence (Table 1). To evaluate the relationships among HDS sequences, we conducted pairwise alignment analysis on their amino acid sequences. It is important to highlight that partial HDS sequences were excluded from this analysis, due to their insufficient length for meaningful alignment. Thus, we focused on analyzing full‐sequence HDS, encompassing HDS12, HDS13, HDS15, HDS17, HDS18, HDS19, HDS20, HDS21, HDS22, HDS26, HDS27, HDS28, HDS30, HDS31, HDS32, HDS34, and HDS35. Figure 2 provides a visual representation of sequence similarity or divergence, offering insights into the evolutionary and functional relationships among the HDSs. Remarkably, the analysis of the entire set of HDS sequences reveals that a significant majority have a similarity of over 30%. Notably, this similarity predominantly manifests within conserved motifs spanning the sequences, mainly the hinge region in the C terminus (P17‐P9) and the S3a domain (NAVYFKG) motif in the N‐terminus (alignment not shown). Beyond the RCL and other conserved regions, extensive sequence divergence was observed in the remaining portions of the sequences. More interestingly, significant levels of similarity were observed among subsequent pairs of sequences: 97.89% for HDS30‐HDS31, 99.5% for HDS28‐HDS27, 85.21% for HDS21‐HDS22, and 74.5% for HDS17‐HDS18.

**Table 1:** Biochemical features of HDSs based on their predicted amino acid sequences.

HDS ID	Male (M) or female (F) expression	ORF cDNA sequence (bp)	Full‐length amino acid residues (aa)	Signal peptide (aa)	Molecular weight (Da)	Glycosylation site		Predicted P1 site	Predicted target
						N glycosylation	O glycosylation		
HDS12	M/F	1209	403	SP (21)	43,159.27	N_88_	T_184_; S_293_; S_339_; S_342_; S_345_	K^a^	Trypsin‐ or thrombin‐like proteases
HDS13	M/F	1134	377	NSP	42,206.64	N_71_;N_304_	S_287_;T_295_	C^b^	Elastase‐ or chymotrypsin‐like proteases
HDS15	M/F	1161	378	NSP	40,797.09	N_23_;N_350_	S_184_	C^b^	Elastase‐ or chymotrypsin‐like proteases
HDS17	M/F	1140	377	NSP	41,524.91	—	S_189_	K^a^	Trypsin‐ or thrombin‐like proteases
HDS18	M/F	1061	353	NSP	38,655.57	N_215_	—	K^a^	Trypsin‐ or thrombin‐like proteases
HDS19	M/F	1413	379	NSP	42,439.62	N_51_	—	C^b^	Elastase‐ or chymotrypsin‐like proteases
HDS20	M/F	1224	403	SP (17)	44,813.71	N_130_; N_231_	—	R^a^	Trypsin‐ or thrombin‐like proteases
HDS21	M/F	1209	399	SP (17)	43,456.79	N_107_; N_258_	T_203_; T_204_; T_316_	I^c^	Not specified
HDS22	M/F	1209	399	SP (17)	43,157.47	N_107_; N_258_	T_203_	L^c^	Not specified
HDS26	M/F	1290	415	NSP	45,464.03	N_333_	S_31_; S_222_; S_328_;T_329_	S^b^	Elastase‐ or chymotrypsin‐like proteases
HDS27	M/F	1236	407	SP (30)	45,199.38	N_99_; N_146_; N_317_	—	R^a^	Trypsin‐ or thrombin‐like proteases
HDS28	M/F	1230	398	NSP	44,112.20	N_90_; N_137_; N_308_	—	R^a^	Trypsin‐ or thrombin‐like proteases
HDS30	M/F	1179	380	NSP	42,423.74	N_55_; N_308_	T_186_; T_299_	Y^b^	Elastase‐ or chymotrypsin‐like proteases
HDS31	M/F	1176	380	NSP	42,394.75	N_55_; N_308_	T_186_; T_299_	Y^b^	Elastase‐ or chymotrypsin‐like proteases
HDS32	F	1236	400	SP (17)	43,446.05	N_108_	T_88_; T_204_	T^b^	Elastase‐ or chymotrypsin‐like proteases
HDS34	M/F	1161	376	NSP	41,414.67	N_115_	T_182_; S_183_;S_189_;S_190_	R^a^	Trypsin‐ or thrombin‐like proteases
HDS35	M/F	1065	336	NSP	36,889.74	N_48_; N_101_; N_207_	S_34_; S_143_	K^a^	Trypsin‐ or thrombin‐like proteases

*Note:* M: male; F: female; SP: signal peptide is present.

Abbreviation: NSP, no signal peptide.

^a^Polar basic.

^b^Polar uncharged.

^c^Hydrophobic.

Table 1 provides detailed insights into the biochemical characteristics of each HDS, as inferred from their predicted amino acid sequences. Each of the full‐length cDNA sequences encoding HDS12, HDS20, HDS21, HDS22, HDS27, and HDS32 contains a single ORF, coding for proteins with signal peptides, suggesting that these proteins are secreted and function extracellularly (Table 1). The remaining sequences lacking a signal peptide are likely to undertake intracellular functions within the salivary gland cells. Moreover, our in silico analysis revealed that all putative full‐length HDSs exhibit potential glycosylation sites (Table 1). The predicted targets for each HDS were identified as described in Table 1, based on their predicted P1 sites and relevant literature.

### 3.3. Each HDS Possess Unique RCL Sequences With Diverse Amino Acid Residues at the P1 Sites

Since the RCL serves as the functional signature of serpins and offers insight into their inhibitory activity [5], our intraspecies analysis specifically targets this region. Figure 3 illustrates the multiple sequence alignment of the 17 RCL sequences extracted from the full‐length HDSs. These RCL sequences extend from P17 to P4′, are similar to those of other serpins, comprising 21 amino acids, and are situated near their C‐terminus (Figure 3). P′ residues are numbered from the cleavage site to the C‐terminus and P residues are numbered from the cleavage site to the N‐terminus. The majority of consensus critical residues for inhibitory activity have been preserved across all RCL sequences, including P17 [E], P15 [G], P14 [T/S], and P12 [A]. However, P16 and P9 were not conserved in serpins HDS15, HDS12, HDS20, HDS21, HDS26, HDS27, HDS28, and HDS31, respectively. Within the RCL sequence lies the scissile P1–P1’ bond, also referred to as the cleavable reactive center, where proteases execute cleavage and form a covalent complex with the serpin [1]. Based on molecular analysis predictions, it is admitted that the cleavage site is located 17 amino acid residues between the beginning of the RCL hinge region and the scissile bond. In accordance with these conventions, we have observed a diversity of 8 P1 residues across the analyzed sequences. Initial manual inspection of RCLs revealed significant similarities between sequences exhibiting identical charge and polarity at the predicted P1 site, leading us to categorize them into three main groups: polar basic, polar uncharged, and hydrophobic P1 residues. Overall, the majority of HDS (47.06%) exhibit polar basic residues, followed by polar uncharged (41.18%), with hydrophobic residues being the least prevalent (11.76%) at the P1 position.

Figure 3Multiple sequence alignment of the HDS reactive center loops. (a) The consensus critical residues for inhibitory activity have been described elsewhere [22]. P notation was applied according to a previous study (Schechter and Berger, 1967). The asterisk indicates positions which have a single, fully conserved residue. (b) The WebLogo depicting the multiple sequence alignment of the RCL region of the 17 protein sequences. The logo shows the conservation of various residues. The *y*‐axis represents the bit score. A score of 4 on *y*‐axis means 100% conservation. The *x*‐axis displays the position of amino acids in the multiple sequence alignment.

**Figure:**
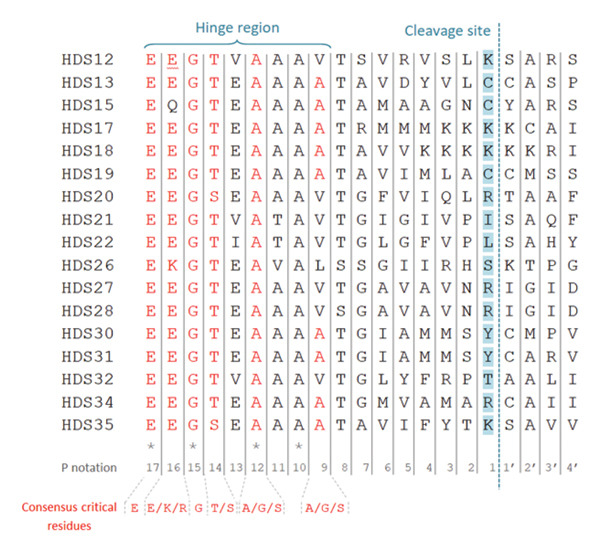
(a)

**Figure:**
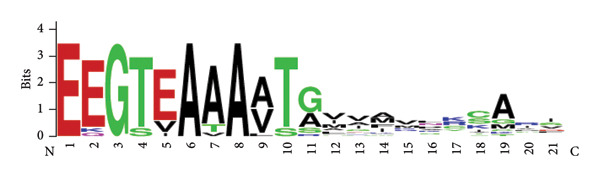
(b)

### 3.4. Some HDSs Are Highly Identical to Their Orthologs in Other Tick Species

**Figure 4:**
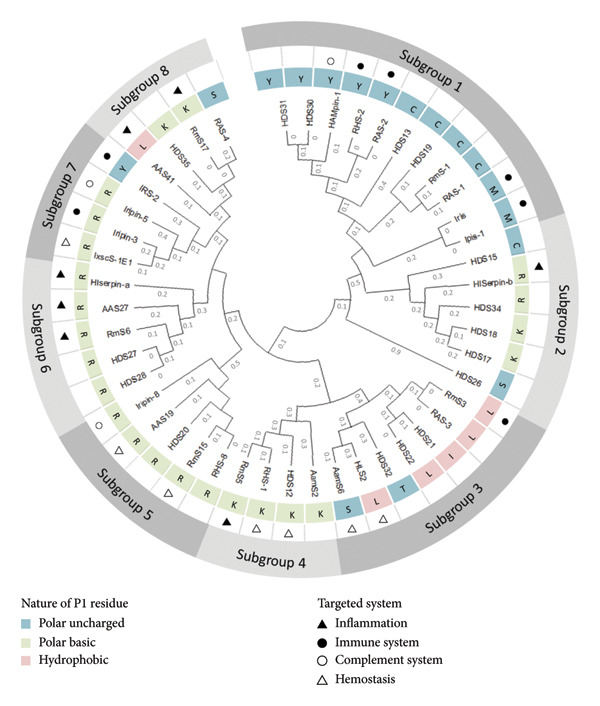
Phylogenetic tree and P1 site residues of HDSs and 29 serpins from other tick species. The evolutionary history was inferred by using the maximum likelihood method and JTT matrix‐based model [20]. The tree with the highest log likelihood (−20571.35) is shown. This analysis involved 46 full‐length amino acid sequences of the serpins. Multiple alignments and evolutionary analyses were conducted in MEGA11 [21].

To study the interspecies relationships between HDSs and other serpins from various tick species, a phylogenetic analysis was performed (Figure 4). The sequences of 28 tick serpins retrieved from eight different tick species were collected from publicly available databases. Table 2 provides the accession numbers of these sequences, along with the percentage identity and coverage with the different HDSs. The phylogenetic tree in Figure 4 was constructed using the full‐length amino acid sequences of each serpin. Our results showed a high sequence identity among some serpins from *H. dromedarii* and their orthologs in other tick species (≥ 60%–91%, see Table 2) highlighting an evolutionary conservation.

**Table 2:** GenBank accession numbers of tick serpins used in the phylogenetic analysis and their best identity with HDS sequences.

Serpin	Tick species	Accession number	Best identity with HDS			References
			HDS	Identity (%)	Coverage (%)	
AamS2	*A. americanum*	ABS87354.1	HDS12	60.71	99	[23]
AamS6	*A. americanum*	ABS87358.1	HDS22	70.18	100	[23]
AAS19	*A. americanum*	JAI08902.1	HDS20	84.86	95	[24]
AAS27	*A. americanum*	JAI08961.1	HDS27	71.86	100	[24]
AAS41	*A. americanum*	JAI08957.1	HDS35	78.23	100	[25]
HLS2	*Hae. longicornis*	BAD11156.1	HDS22	68.67	100	[24]
HlSerpin‐a	*Hae. longicornis*	QFQ50847.1	HDS28	65.91	100	[26]
HlSerpin‐b	*Hae. longicornis*	QFQ50848.1	HDS34	58.89	99	[27]
HAMpin1	*H. anatolicum*	—	HDS31	96.85	100	[14]
Ipis‐1	*I. persulcatus*	BAP59746.1	HDS30	60.3	98	[27]
Iripin‐3	*I. ricinus*	JAA69032.1	HDS27	46.75	100	[28]
Iripin‐5	*I. ricinus*	—	HDS28	47.50	100	[29]
Iripin‐8	*I. ricinus*	ABI94058.1	HDS20	77.67	100	[30]
Iris	*I. ricinus*	CAB55818.2	HDS31	60.05	98	[31]
IRS‐2	*I. ricinus*	ABI94056.2	HDS27	47.25	98	[32]
IxscS‐1E1	*I. scapularis*	AID54718.1	HDS27	46.87	100	[33]
RAS‐1	*Rh. appendiculatus*	—	HDS19	73.95	99	[12]
RAS‐2	*Rh. appendiculatus*	AAK61376.1	HDS31	81.02	96	[34]
RAS‐3	*Rh. appendiculatus*	AAK61377.1	HDS22	82.46	100	[34]
RAS‐4	*Rh. appendiculatus*	AAK61378.1	HDS35	71.88	64	[34]
RmS‐1	*Rh. microplus*	AHC98652.1	HDS19	79.42	99	[35]
RmS3	*Rh. microplus*	AHC98654.1	HDS22	84.46	100	[35]
RmS‐6	*Rh. microplus*	AHC98657.1	HDS28	79.45	100	[35]
RmS‐5	*Rh. microplus*	AHC98656.1	HDS12	80.45	100	[35]
RmS‐15	*Rh. microplus*	AHC98666.1	HDS20	88.59	100	[35]
RmS‐17	*Rh. microplus*	AHC98668.1	HDS35	88.39	85	[35]
RHS‐1	*Rh. haemaphysaloides*	AFX65224.1	HDS12	83.33	99	[36]
RHS‐2	*Rh. haemaphysaloides*	AFX65225.1	HDS31	87.11	100	[36]
RHS‐8	*Rh. haemaphysaloides*	QHU78941.1	HDS20	91.07	100	[36]

Our analysis clustered a total of 46 serpins into three main groups, which are further divided into eight subgroups (Figure 4). These serpins have been grouped based on their sequence similarities and shared functional motifs, particularly focusing on the P1 residue which is crucial for determining their inhibitory specificity. By examining the evolutionary trajectory of serpins in *H. dromedarii*, we aim to understand how these proteins have diversified and adapted over time. For instance, subgroup 1 includes HDS19, HDS13, HDS31, and HDS30, which cluster together with serpins from the *Rhipicephalus* genus and one serpin from *Hyalomma* genus. This subgroup is characterized by serpins that have a polar uncharged amino acid at the P1 site. Similarly, other tick serpins in this subgroup (Iris, Ipis1, RmS1, RAS1, RHS2, HAMpin1, and RAS2) also feature a polar uncharged amino acid at the P1 site and have been described as targeting immune system components, playing crucial roles in modulating host immune responses. Likewise, HDS12 from subgroup 4 and HDS20 from subgroup 5 have a polar basic amino acid at the P1 site, similar to their orthologs in their respective subgroups. Some HDSs are suspected to modulate host inflammation, including HDS35 from subgroup 8, HDS27 and HDS28 from subgroup 6, and HDS17, HDS18, HDS26, HDS15, and HDS34 from subgroup 2. The remaining HDSs (HDS22, HDS21, HDS15, and HDS26) are likely to target a broader spectrum of proteases involved in several physiological pathways.

## 4. Discussion

Serpins are abundant in tick secretions and have mostly immunological and hemostatic functions [37]. Traditionally, the characterization of tick serpins has relied heavily on laborious experimental techniques [38]. However, recent advancements in bioinformatics have revolutionized our ability to explore and analyze large‐scale genomic and proteomic datasets, providing unprecedented insights into the molecular intricacies of tick–host interactions [39, 40]. In this context, bioinformatics tools offer a powerful means to predict, annotate, and characterize serpins encoded within tick genomes, enabling us to uncover hidden relationships and functionalities. In the current study, we leverage the valuable insights derived from a prior research describing the sialotranscriptome of both genders of the camel tick, *Hyalomma dromedarii* [18], and delve deeper into the nuanced characteristics of serpins expressed in this tick species.

This investigation is the first thorough examination of serpins expressed in the sialotranscriptome of *H. dromedarii*, revealing a count of 110 distinct transcripts coding for these proteins. Since the publication of the first tick genome of *Ixodes scapularis* [41], several tick transcripts coding for serpins have been documented. The *I. scapularis* genome counted 45 serpins [42, 43], while 36 serpins were found in the sialotranscriptome of *I. ricinus* [44]. Numerous reports have also highlighted transcripts coding for serpins from the *Amblyomma* genus, including 36 serpins in *A. maculatum* [45], 50 in *A. sculptum* [46], and only one coding sequence in *A. tuberculatum* [47]. Additionally, 22 distinct transcripts coding for serpins were discovered in *Rhipicephalus microplus* [48] and 8 in *Rh. sanguineus* [49], whereas only 10 were noted in *H. excavatum* [50]. In comparison to these previous studies, our investigation reveals a relatively high number of serpins expressed in *H. dromedarii.* This high number is in line with reports from *A. americanum*, which showed 120 serpin transcript counts [51]. This diversity of serpin transcripts may be linked to the specific roles these proteins play in immune response and blood‐feeding. Indeed, as a hard tick, *H. dromedarii* has an extended feeding period, which likely requires regulators, such as serpins, to inhibit host defense systems. To validate the predicted functions of HDSs, further experimental studies are necessary.

Our data revealed a greater number of serpin transcripts in male ticks compared to females (106 vs. 95 transcripts). This finding aligns with previous reports that have also noted a higher abundance of serpin transcripts in male ticks [24]. Indeed, in that previous study, they counted 57 *A. americanum* serpin (AAS) transcripts in male and 33 in female ticks, with 30 transcripts being common to both genders. This difference in the number of serpin transcripts and their expression level between male and female ticks suggests potential variations in their physiological roles and responses to environmental stimuli. In fact, this expression pattern suggests potential involvement in sex‐specific processes such as reproduction, development, or aiding the tick during feeding. Serpins play crucial roles in regulating proteolytic processes, which are vital for tick survival, development, and reproduction [37]. Thus, their higher expression in male ticks might be linked to their specific biological functions, such as spermatogenesis, mating behavior, or other sex‐specific physiological processes [52]. Controversially, when we examined on a per‐transcript basis, some female transcripts showed significantly higher expression levels compared to males, according to the heatmap of HDSs’ gene expression levels (Figure 1(b)). These overexpressed HDSs in females may directly contribute to reproductive processes, such as egg development and vitellogenesis, by regulating protease activities. Additionally, they may also have indirect roles, as female ticks require significant amount of blood for egg production, resulting in longer feeding durations compared to males [53]. Abundant HDSs in the saliva of female *H. dromedarii* might play a crucial role in modulating the host’s defense response to facilitate prolonged feeding. Overall, the heatmap of HDSs’ gene expression levels in female and male *H. dromedarii* may provide valuable insights into the transcriptional dynamics of these genes and their potential roles in sex‐specific processes within the tick species. Further investigation into these clusters may provide deeper insights into the molecular mechanisms governing tick biology and physiology.

According to our in silico analysis, overall features of the serpins from *H. dromedarii* tick salivary glands are consistent with previously described tick serpins [37]. Typically, serpins are around 45 kDa and are relatively large molecules (∼350‐400 amino acids) compared to other protease inhibitors [5]. The presence of signal peptide in most of the HDS sequences is indicative of secretion and extracellular activity. Although there is no direct evidence documenting the secretion of these serpins at the bite site, there is a potential that this may be the case given the inhibitory function of both platelet aggregation and blood coagulation of HDS12 (Dromaserpin) [10]. It is also notable that despite the presence of several glycosylation sites, HDSs may not require glycosylation for their functionality, as they belong to the serpin superfamily. Indeed, several studies have reported the inhibitory functions of nonglycosylated *E. coli-*expressed serpins, suggesting that glycosylation may not be essential to serpin function [27, 54, 55]. Similarly, when expressed in vitro, HDS12 (rDromaserpin) showed functional nonglycosylated recombinant protein in agreement with these previous investigations [10]. The presence of glycosylation sites in these HDSs may hint at additional regulatory mechanisms or structural characteristics beyond conventional glycosylation‐dependent functions. Further investigation into the role of glycosylation in HDSs could provide valuable insights into their biological functions and regulatory mechanisms within the context of salivary gland physiology.

The RCL stands as the functional signature of serpins, with its amino acid sequence offering valuable clues regarding their inhibitory capabilities [5]. Notably, when comparing RCL sequences in HDSs, a significant similarity predominantly manifests within conserved motifs spanning the sequences, mainly the hinge region in the C terminus (P17‐P9) and the S3a domain (NAVYFKG) motif in the N‐terminus (alignment not shown). This observation underscores the presence of conserved regions across the HDS sequences, indicating potential functional similarities and suggesting a common evolutionary origin. Although several HDS pairs exhibit overall sequence identity values below 40%, it is well established in serpin comparative studies that functional conservation is often retained despite low global sequence identity. This is because serpin activity is primarily governed by the conservation of key structural and regulatory regions rather than overall sequence similarity. In contrast, certain HDS pairs demonstrate notably high similarity, with identity values of 97.89% for HDS30–HDS31, 99.5% for HDS28–HDS27, 85.21% for HDS21–HDS22%, and 74.5% for HDS17–HDS18. These findings suggest the presence of sections in their sequences that are entirely similar, apart from the conserved regions, indicating that they are likely to be isoforms or variants from the same or different gene (*s*).

Within the RCL sequence lies the scissile P1–P1’ bond, also known as the cleavable reactive center, where the protease cleaves and forms a covalent complex with the serpin [1]. According to previous structural studies, this bond could determine the protease selectivity and specificity [56]. Although the target protease of a serpin cannot be predicted precisely based only on its P1 amino acid residue, various studies have correlated the nature of P1 residue with the target protease [57, 58]. In this study, each HDS has a distinct RCL sequence, all featuring a diverse array of eight amino acid residues at the P1 sites, with the majority (47.06%) having polar basic residues. In the literature, it is generally agreed that serpins targeting trypsin or thrombin‐like proteases possess polar basic (Arg and Lys) residues at the P1 site, leading to the hypothesis that these serpins interact with blood‐coagulation proteases [59]. This hypothesis aligns with the predicted targets of HDS12, HDS17, HDS18, HDS20, HDS27, HDS28, HDS34, and HDS35, which are likely involved in antihemostatic activities. These HDSs might inhibit host blood clotting during feeding by targeting specific serine proteases involved in the coagulation cascade, ensuring a continuous flow of blood essential for ticks to obtain nutrients [53, 60]. Their predicted ability to target trypsin‐ and thrombin‐like proteases further supports the need for in vitro investigation. Notably, HDS12, previously described as rDromaserpin, has been tested in vitro and has been described as a potential candidate for developing therapeutic compounds targeting disorders related to blood clotting and platelet aggregation [10]. In other hands, evolutionary analysis suggests that some HDSs may also modulate host inflammation and the immune system, while others are thought to target a broader spectrum of proteases involved in various physiological pathways. Indeed, HDSs with aromatic (Phe, Tyr, and Trp) residues at the P1 site are more likely to inhibit chymotrypsin‐like proteases [61]. Serpins with neutral (mainly Met, Ser, Ala, Val, Ile, and Leu), inhibiting both trypsin and chymotrypsin, are interesting though, but not unusual [62]. These proteins might interact with various components of the host immune system, possibly inhibiting key proteases involved in immune responses. This immunomodulatory function could be beneficial for the tick, helping it evade host immune defenses and facilitating successful feeding and pathogen transmission. From a drug discovery perspective, these inhibitors could serve as promising therapeutic agents for treating hemostatic disorders [37] or inflammation [13]. Future research should focus on experimentally validating these predictions and exploring their potential applications in controlling tick‐borne diseases or in drug discovery development.

## 5. Conclusion

In conclusion, this paper provides a comprehensive bioinformatic analysis of serpins expressed in *H. dromedarii* salivary glands, highlighting their potential roles in tick–host interactions. Integrating bioinformatics with experimental validation will be crucial for uncovering the specific functions of these serpins in pathogen transmission, immune modulation, and hemostasis. Approaches such as gene expression profiling, recombinant protein characterization, functional inhibition assays, and animal models will enable a detailed understanding of HDS inhibitory mechanisms and their contributions to tick physiology. Elucidating these mechanisms may have significant implications for the development of novel antitick interventions, including therapeutics and vaccines. Continued interdisciplinary research will be vital for harnessing the therapeutic and vaccine potential of HDSs [63].

## Conflicts of Interest

The authors declare no conflicts of interest.

## Funding

This research was funded by Institut Pasteur de Tunis‐Laboratory Viruses, Vectors and Hosts (LR20IPT02). Fernanda Faria received a funding from Fundação Butantan.

## Data Availability

The data that support the findings of this study are available from the corresponding author upon reasonable request.
